# Homœopathy Phrenologically Considered

**Published:** 1890-01

**Authors:** Geo. W. Sherbino

**Affiliations:** Abilene, Texas


### HOMŒOPATHY PHRENOLOGICALLY CONSIDERED.

GEO. W. SHERBINO, M. D., ABILENE, TEXAS.

I. The organ of amativeness is influenced by Can-ind., Canth., Conium, Hyos., Kali-brom., Lach., Lilium-tig., Murex-p., Nux-vom., Phos., Phos-acid., Sabina, Staphis., Stram., Verat-a., and others.

II. Combativeness, by Acon., Agar-m., Alco., Amb., Ant-t., Arsen., Aur., Bar-c., Bell., Brom., Bry., Calc-c., Canth., Caust., Cham., China, Elaps, Ferr., Hyos., Ig., Kali-i., Lach., Lycopod., Nux-vom., Petrol., Platina, Ruta, Sep., Staph., Stram., Sul-ac., Tarent., Thuja, Verat-v., Viola-t., Zn.

III. Destructiveness, Bell., Hyos., Mer-i-f., Opium, Veratrum-alb.

IV. Acquisitiveness, Bry-a., Calc-c. (Calc-flu.), Nux-v., Puls.
V. Firmness, Bell., Calc-c., Cham., Lyco., Nux-v., Nitric acid, Sanicula, Silicea, Stram., Sulph.
VI. Caution, Aconite, Alcoh., Arsen., Cup-m., Hyos., Nux-v., Opium, Puls., Stram.
VII. Benevolence, Alcoh., Anacard., Coff-t.
VIII. Conscientiousness, Arsen., Aurum, Ign., Hyos., Nux-v., Sil.
IX. Hope, Acon., Calc-c., Ferr-m. (alternating with sadness, Raph.) Sulph., Verat-a.
X. Marvellousness, Stramonium.
XI. Imitation, Stram.
XII. Form, Bell., Cup., Hyos.
XIII. Causality, Stram.
XIV. Veneration, Coff.
XV. Secretiveness, Agar-m., Alco.
XVI. Mirth, Acon., Ænanth., Arum., Asaf., Bell., Crocus, Hyos., Ign., Nux-m., Puls., Phos., Sepia, Stram.
XVII. Adhesiveness, Bell., sweet outpourings of Alco.
XVIII. Self-Esteem, Ign. and Platina.
XIX. Philoprogenitiveness, Ox-ac.

